# IRWRLDA: improved random walk with restart for lncRNA-disease association prediction

**DOI:** 10.18632/oncotarget.11141

**Published:** 2016-08-09

**Authors:** Xing Chen, Zhu-Hong You, Gui-Ying Yan, Dun-Wei Gong

**Affiliations:** ^1^ School of Information and Electrical Engineering, China University of Mining and Technology, Xuzhou, 221116, China; ^2^ School of Computer Science and Technology, China University of Mining and Technology, Xuzhou, 221116, China; ^3^ Academy of Mathematics and Systems Science, Chinese Academy of Sciences, Beijing, 100190, China; ^4^ National Center for Mathematics and Interdisciplinary Sciences, Chinese Academy of Sciences, Beijing, 100190, China

**Keywords:** lncRNAs, disease, cancer, random walk with restart

## Abstract

In recent years, accumulating evidences have shown that the dysregulations of lncRNAs are associated with a wide range of human diseases. It is necessary and feasible to analyze known lncRNA-disease associations, predict potential lncRNA-disease associations, and provide the most possible lncRNA-disease pairs for experimental validation. Considering the limitations of traditional Random Walk with Restart (RWR), the model of Improved Random Walk with Restart for LncRNA-Disease Association prediction (IRWRLDA) was developed to predict novel lncRNA-disease associations by integrating known lncRNA-disease associations, disease semantic similarity, and various lncRNA similarity measures. The novelty of IRWRLDA lies in the incorporation of lncRNA expression similarity and disease semantic similarity to set the initial probability vector of the RWR. Therefore, IRWRLDA could be applied to diseases without any known related lncRNAs. IRWRLDA significantly improved previous classical models with reliable AUCs of 0.7242 and 0.7872 in two known lncRNA-disease association datasets downloaded from the lncRNADisease database, respectively. Further case studies of colon cancer and leukemia were implemented for IRWRLDA and 60% of lncRNAs in the top 10 prediction lists have been confirmed by recent experimental reports.

## INTRODUCTION

For quite a long time, genetic information was considered to be only stored in protein-coding genes and RNA was just transcriptional noise and intermediary between a DNA sequence and its encoded protein [1–5]. However, sequence analysis indicates that ~ 20,000 protein-coding genes in the human genome make up only a very small fraction (approximately 1.5%) of the complete human genome [6–12]. In other word, more than 98% of the human genome does not encode protein sequences, yielding tens of thousands of non-coding RNAs (ncRNAs). Further studies demonstrate that the proportion of non-protein-coding sequences would significantly increase with organism complexity and ncRNAs have been confirmed to play very critical regulation roles in various fundamental and important biological processes [13, 14]. According to the transcript lengths, ncRNAs could be further divided into two categories: small ncRNAs (transcripts with the length shorter than 200 nucleotides, such as microRNA (miRNA), transfer RNA, and Piwi interacting RNA) and long ncRNAs (lncRNA, transcripts with the length more than 200 nucleotides) [11, 15–17]. The functional features of protein-coding genes and lncRNAs are significantly different. LncRNAs have less conservation across species, much more tissue-specific pattern, and relatively lower expression level than protein-coding genes [4, 18, 19]. Therefore, it is no surprise that lncRNAs were considered to be transcriptional noise when they were first discovered [3, 4]. The first two lncRNAs, H19 and Xist, were discovered based on traditional gene mapping approaches in the early 1990s [20–22]. In the recent several years, with the rapid development of high-throughput sequencing technologies and computational models, researchers have identified a large number of lncRNAs in eukaryotic organisms ranging from nematodes to humans [19, 23]. For example, Guttman et al. (2009) integrated gene expression data, the presence of chromatin marks for promoter regions and gene bodies, and the known annotations of coding transcripts to propose the first large-scale lncRNA discovery approach. In their studies, 1600 novel mouse large intervening non-coding RNAs (lincRNAs) across four mouse cell types have been discovered [23]. Another important example of lncRNA discovery is that Cabili et al. (2011) integrated chromatin marks and RNA-sequencing (RNA-seq) data to identify more than 8000 lincRNAs across 24 different human cell types and tissues [24].

In recent years, accumulating evidences have shown that lncRNAs play critical regulation roles in various fundamental and vital biological processes, including cell differentiation, proliferation and apoptosis, cell cycle control, cellular transport, transcriptional and post-transcriptional regulation, epigenetic regulation, organ or tissue development, and tumorigenesis [3, 12, 23, 25–27]. This important discovery has ended the arguments on lncRNA functions and disabused the false viewpoint that the lack of conservation would imply lack of functions [4, 28–30]. Complex secondary and tertiary structures of lncRNAs allow them to modulate the action of proteins or miRNAs [28, 31]. Furthermore, other studies revealed that lncRNAs could regulate protein-coding genes in oncogenic and tumor suppressive pathways at transcriptional, posttranscriptional, and epigenetic levels [32]. Experimental studies in the melanoma cell lines indicated that lncRNA sprouty homolog 4 intronic transcript 1 (SPRY4-IT1) could inhibit cell proliferation and apoptosis [33]. However, the exact mechanism behind lncRNA-related regulation interactions still remains unclear for most of the lncRNAs [28]. Recently, with the development of novel experimental and computational approaches, the dysregulations of lncRNAs are confirmed to be associated with a wide range of human diseases, such as many types of cancers [34, 35], neurodegenerative disorders [36], cardiovascular diseases [37], and diabetes [38]. Especially, some specific lncRNAs have been found to be associated with various diseases. For example, the dysfunction of lncRNA H19 is associated with various cancers, such as Breast cancer, Lung cancer, Colon cancer, Gastric cancer, Liver cancer, and Bladder cancer [3]. Based on a knockdown approach, down-regulation of H19 could significantly decrease the clonogenicity and anchorage-independent growth of breast and lung cancer cell [39]. For the bladder cancer, H19 has been considered as a potential prognostic biomarker for the early recurrence [40]. Recent studies also indicated that H19 could enhance carcinogenesis and metastasis of gastric cancer through the direct upregulation of ISM1 and the indirect suppression of CALN1 expression [41].

Nowadays, more than 15,000 human lncRNAs have been annotated and stored in GENCODE [30]. However, a relatively limited number of lncRNAs have been extensively studied to shed light on their functions and potentially associated diseases. Therefore, identifying disease-related lncRNAs is still a great challenge. The identification of lncRNA-disease associations could not only benefit better understanding of underlying mechanism causing various human diseases at lncRNA level, but also accelerate potential biomarker identification for disease diagnosis, treatment, prognosis, and drug response prediction [42–45]. The study of identifying novel lncRNA–disease associations has attracted the attentions of more and more researchers and become one of the most important topics in the research fields of lncRNAs and diseases. Considering the increasing number of newly discovered lncRNAs each year, identifying disease-lncRNA associations based on biological experiments has encountered many bottlenecks due to experimental time and cost. On the other hand, the detailed information of lncRNA sequence, expression, function has been also collected by some publicly available databases, such as NRED [46], lncRNAdb [25], NONCODE [26]. Therefore, it is necessary and feasible to analyze known lncRNA-disease associations, predict potential lncRNA-disease associations, and provide the most possible lncRNA-disease pairs for experimental validation. Computational models could significantly decrease the time and cost of biological experiments by quantifying the association probability of each lncRNA-disease pair [47–51].

Some computational methods have been developed to predict potential disease-lncRNA associations. In 2013, Chen et al. developed a reliable computational tool of LRLSLDA to predict novel human lncRNA-disease associations based on the assumption that similar diseases tend to be related with functionally similar lncRNAs. This model is mainly based on a semi-supervised learning framework of Laplacian Regularized Least Squares [48], which integrates known disease–lncRNA associations and lncRNA expression profile. Therefore, LRLSLDA doesn't need the information of negative samples, which are difficult to obtain. However, this model has an intrinsic problem that two different classifiers were obtained in the disease space and lncRNA space, respectively. How to directly obtain a single classifier or reasonably combine these two classifiers is not yet completely solved. In 2015, based on the assumption that similar diseases tend to be associated with lncRNAs with similar functions, Chen et al. further developed two novel lncRNA functional similarity calculation models (LNCSIM) [52]. In the model of LNCSIM, disease semantic similarity was first calculated based on the directed acyclic graph (DAG) which represents the relationships among different diseases. Then, lncRNA functional similarity was further obtained by calculating the semantic similarity between their associated disease groups. When LNCSIM and LRLSLDA were further combined, the predictive performance was improved in both cross validation and case studies of colorectal cancer and lung cancer. However, how to select semantic contribution decay factor is worthy of further research. Based on the same assumption, Sun et al. developed a global network similarity-based computational model of RWRlncD to identify potential lncRNA-disease associations by implementing classical random walk with restart (RWR) on the constructed lncRNA functional similarity network [53]. RWRlncD integrates known lncRNA-disease association networks, disease similarity network, and lncRNAs functional network. However, RWRlncD can only be applied to the lncRNAs with known related diseases, limiting the wide application of RWRlncD. In the same framework of RWR, Zhou et al. developed the model of RWRHLD to predict the potential lncRNA-disease associations by implementing random walk on the heterogeneous network constructed by the integration of lncRNA-lncRNA crosstalk network, disease-disease similarity network, and known lncRNA-disease association network [54]. Specially, lncRNA crosstalk network was constructed based on the assumption that similar lncRNAs tend to share significantly interacting miRNAs. Therefore, RWRHLD cannot be applied to the lncRNAs without known lncRNA-miRNA interactions. Yang et al. implemented a propagation algorithm on the coding-non-coding gene-disease bipartite network to infer potential lncRNA-disease associations [55]. The coding-non-coding gene-disease bipartite network was constructed by integrating known lncRNA-disease associations and gene-disease associations. However, this computational model cannot be applied to the diseases without any known related genes. Recently, the computational model of KATZLDA was developed to identify potential lncRNA-disease associations by known lncRNA-disease associations and various similarity measures of diseases and lncRNAs [56]. More importantly, this new model could be applied to diseases without any known related lncRNAs and lncRNAs without any known associated diseases.

Considering the fact that known disease-lncRNA associations are still rare, some methods that didn't rely on known lncRNA-disease associations have been developed to infer potential lncRNA-disease associations. For example, Liu et al. integrated human lncRNA expression profiles and gene expression profiles to construct lncRNA-gene co-expression network and identify potential disease-lncRNA associations based on known disease-gene associations and gene-lncRNA co-expression relationships [57]. However, the important limitation of this computational model is that the output of tissue-specific lncRNAs is only tissue-related diseases rather than specific disease names. Li et al. predicted novel associations between vascular disease and lncRNAs based on the genome location [58]. However, some lncRNAs have no neighbor gene within 50 kb distance and the functions of lncRNA are not always related with its neighbor genes. Recently, I have developed the model of HyperGeometric distribution for LncRNA-Disease Association inference (HGLDA) to predict novel lncRNA-disease associations based on the integration of miRNA-disease associations with lncRNA-miRNA interactions. HGLDA obtained the reliable performance in the leave-one-out cross validation (LOOCV) and case studies about breast cancer, lung cancer, and colorectal cancer [49]. However, these three models cannot be applied to the diseases without any known associated genes or miRNAs.

RWR has been widely used in various important problems in computational biology, such as disease-lncRNA association prediction [53, 54], disease-related miRNA identification [59, 60], drug-target interaction inference [61, 62], and disease gene identification [63, 64]. Here, the model of Improved Random Walk with Restart for LncRNA-Disease Association prediction (IRWRLDA) was developed to predict novel lncRNA-disease associations by integrating known lncRNA-disease associations, disease semantic similarity, lncRNA functional similarity, lncRNA expression profile similarity, and lncRNA Gaussian interaction profile kernel similarity. For any given disease, traditional RWR set the initial probability vector only based on known related lncRNAs with this disease. Therefore, RWR cannot work if this disease has no known related lncRNAs. Compared with traditional RWR, the novelty of IRWRLDA lies in the incorporation of lncRNA expression similarity and disease semantic similarity to set the initial probability vector of the RWR. Therefore, IRWRLDA could be applied to diseases without any known related lncRNAs. LOOCV was implemented for evaluating the performance of IRWRLDA based on two versions of lncRNA-disease association datasets (June-2012 Version and June-2014 Version) consisting of known experimentally verified lncRNA-disease associations downloaded from the LncRNADisease database [3]. As a result, IRWRLDA significantly improved previous classical models with reliable AUCs of 0.7242 and 0.7872 in the datasets of June-2012 and June-2014 Version, respectively. Further case studies of colon cancer and leukemia were implemented for IRWRLDA. As a result, 60% of lncRNAs in the top 10 prediction lists were confirmed by recent experimental reports.

## RESULTS

### Performance evaluation

**Figure 1:**
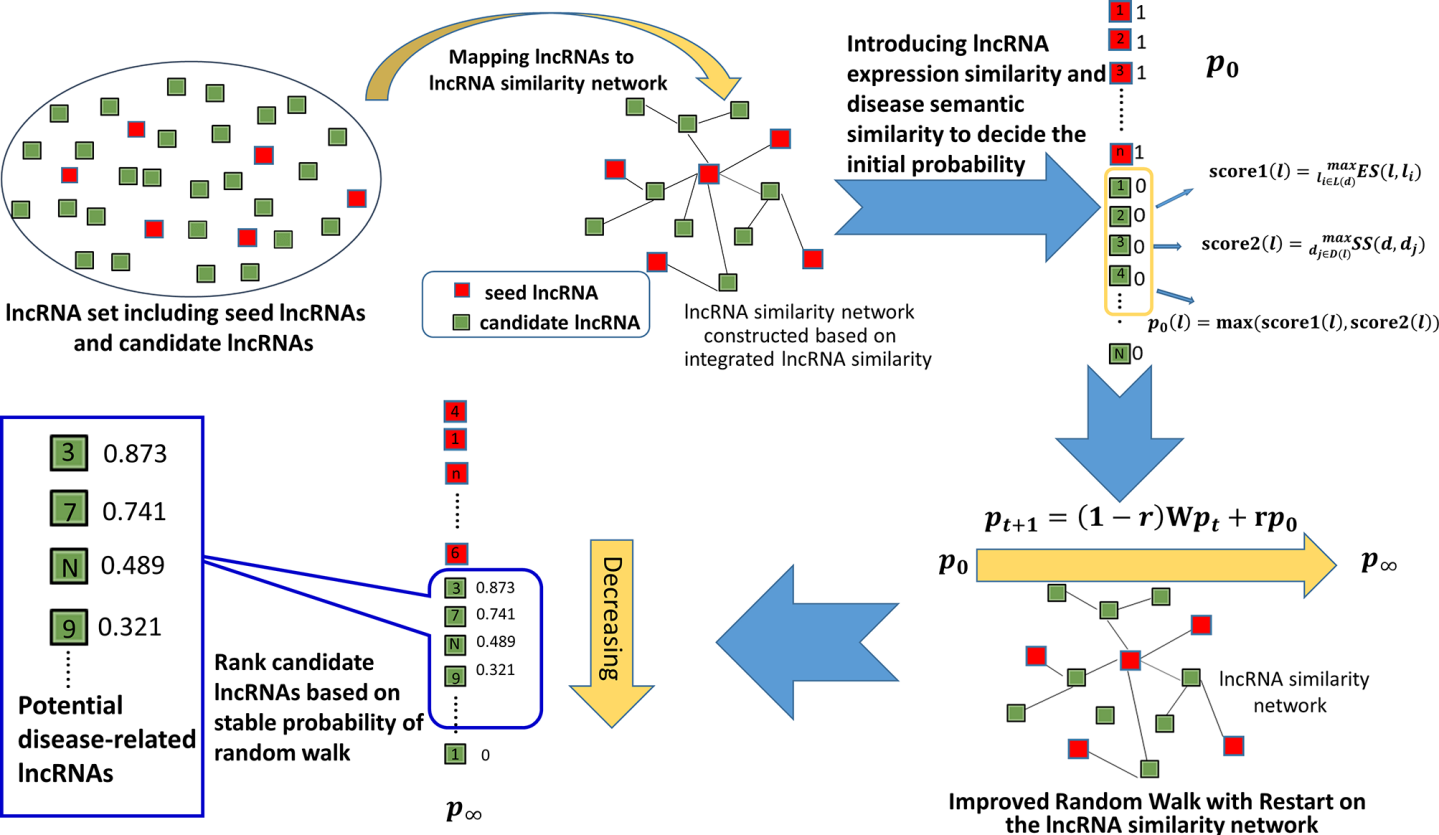
Flowchart of IRWRLDA, demonstrating the basic ideas of uncovering potential disease-lncRNA associations by implementing random walk on the lncRNA similarity network constructed based on integrated lncRNA similarity IRWRLDA consists of the following three steps: (1) decide the initial probability of all the lncRNAs, (2) implement random walk on the lncRNA similarity network constructed based on integrated lncRNA similarity, and (3) obtain stable probability of random walk and rank candidate lncRNAs. The essential difference between IRWRLDA and traditional RWR lies in the initial probability of lncRNAs.

LOOCV was implemented to evaluate the prediction performance of IRWRLDA based on two versions of lncRNA-disease association datasets (June-2012 Version and June-2014 Version) downloaded from lncRNADisease database (see Figure 1 and Methods section for the detail of IRWRLDA). When LOOCV was implemented for the investigated disease, each known related lncRNA was left out in turn as a test sample and other known related lncRNAs were regarded as training samples. All the lncRNAs without known association evidences with the investigated disease were considered as candidate samples. How well the left-out test sample was ranked relative to candidate samples would be evaluated. If the rank of test sample exceeded the given threshold, then it can be considered as a successful prediction. Furthermore, true positive rate (TPR, sensitivity) and false positive rate (FPR, 1-specificity) could be correspondingly obtained according to different thresholds. Sensitivity indicates the percentage of the test samples with ranks higher than the given threshold and specificity indicates the percentage of candidate samples below this threshold. Therefore, Receiver-operating characteristics (ROC) curve can be drawn by plotting TPR versus FPR at different thresholds. Area under ROC curve (AUC) was further calculated to evaluate the performance of IRWRLDA. If AUC = 1, it means perfect performance has been obtained and if AUC = 0.5, it means the prediction result is random.

Comparison between IRWRLDA with other three state-of-the-art disease-lncRNA association prediction models (LRLSLDA [48], RWRlncD [53], and NRWRH [54, 61]) in the framework of LOOCV was implemented. LRLSLDA is the first large-scale lncRNA-disease association prediction model, which has obtained reliable performance in the framework of both cross validation and case studies. Therefore, this model was selected for performance comparison. RWRlncD and NRWRH have been successfully used to predict novel lncRNA-disease associations by independent third party [53, 54]. The comparison between these two models and IRWRLDA could further demonstrate the reasonability of setting the initial probability vector of random walk by lncRNA expression similarity and disease semantic similarity.

**Figure 2:**
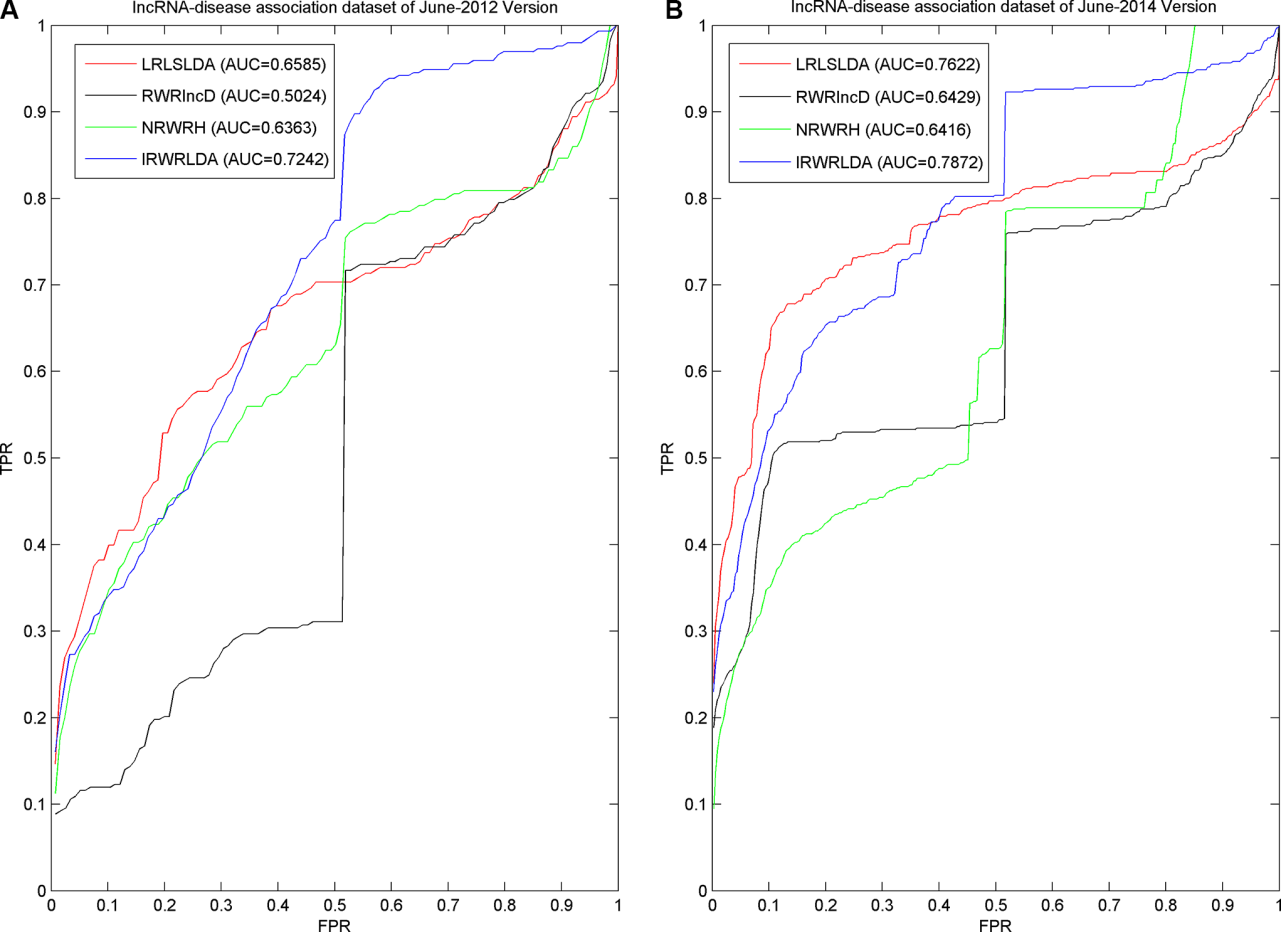
Comparison between IRWRLDA with other three the-state-of-art disease-lncRNA association prediction models in the framework of LOOCV was implemented As a result, IRWRLDA achieved AUCs of 0.7242 and 0.7872 for the June-2012 Version and June-2014 Version datasets, respectively, significantly improving all the previous classical models.

As a result, IRWRLDA achieved AUCs of 0.7242 and 0.7872 for the June-2012 Version and June-2014 Version datasets, respectively (see Figure 2), significantly improving all the previous classical models. RWRlncD can only be applied to the diseases with known related lncRNAs. Therefore, when investigated disease only has 1 known lncRNA, LOOCV cannot be implemented. To be fair, left-out known lncRNA was considered to obtain the random rank in that case, i.e. for N candidate lncRNAs, the rank of left-out known lncRNA was regarded as (N+1)/2. Therefore, huge jumps could be observed on the ROC curves of RWRlncD in Figure 2 at the exact point of FPR = 0.5. IRWRLDA could be successfully applied to new diseases without any known related lncRNAs by integrating lncRNA expression similarity, disease semantic similarity, and known lncRNA-disease associations to jointly decide the initial probability vector of the RWR. When investigated disease only has 1 known lncRNA and this lncRNA is regarded as the test sample to implement LOOCV, the initial probability of lncRNA could be obtained by calculating the maximum of two scores defined in this paper. However, in some cases, the initial probability could be equal to zero for all the lncRNAs, then the left-out known related lncRNAs would again obtain random rank. Therefore, there are still small jumps for the ROC curve of IRWRLDA at the exact point of FPR = 0.5. The same applies for NRWRH, although it also could be applied to disease without known related lncRNAs by regarding the investigated disease as seed node of random walk.

### Case studies

Case studies of colon cancer and leukemia were implemented to further demonstrate reliable prediction performance of IRWRLDA. For the investigated disease, known lncRNA-disease associations in the June-2012 Version dataset were used as training samples and other lncRNAs without known associations with this disease in the June-2012 Version dataset were used as candidate samples. Furthermore, predictive results were validated based on the updates of LncRNADisease database after June, 2012 and recent experimental literatures.

As one of the most common causes of cancer-related deaths worldwide, colon cancer has posed a great threat to global public health. Colon cancer patients and colon cancer deaths increases by 1,200,000 and 600,000 each year, respectively [65–67]. Colon cancer is usually caused by the combination of various factors, such as genetic and epigenetic changes [68, 69]. Specially, lncRNAs have been demonstrated to play a critical role in the development and progression of colon cancer [3]. As a result, six out of top ten potential colon cancer-related lncRNAs predicted by IRWRLDA were confirmed by the updates in the lncRNADisease database [3] and recent biological experiments [70, 71]. For example, top four potentially related lncRNAs (CRNDE, KCNQ1OT1, MALAT1, and HOTAIR) were validated by lncRNADisease database [3]. Recently, Li et al (2015) have investigated the expression of NEAT1 (ranked 5th in the prediction results) in 239 clinical cases of colon cancer specimens and matched normal tissues and demonstrated that NEAT1 may play an oncogenic role in colon cancer differentiation, invasion and metastasis and serve as an important biomarker of colon cancer recurrence and prognosis [70]. Furthermore, according to another experimental literature [71], copy-number alterations of lncRNA GNAS-AS1 (8th in the prediction results) are much frequent in colon cancer samples.

Leukemia is a group of cancers which begin in the bone marrow and cause a large amount of abnormal white blood cells. The major causes of leukemia are the expression changes of protein coding genes and ncRNAs [72]. Recently, further evidences have demonstrated that the mutations and dysregulations of lncRNAs are associated with the development and progression of leukemia [3]. Therefore, IRWRLDA was further implemented to predict leukemia-related lncRNAs. As a result, six out of top ten potentially leukemia-related lncRNAs have been confirmed by the updates in the lncRNADisease database and recent biological experiments [73, 74]. For example, MEG3, WT1-AS, DLEU2 and CDKN2B-AS1 were ranked 1st, 2nd, 4th and 6th, respectively. The associations between these lncRNAs and leukemia were confirmed by the recent updates in the lncRNADisease database. H19 (3rd of the predictive list) was further confirmed to play critical roles in leukemia based on the observation that the loss of imprinting (LOI) of H19 was a frequent event in adult T-cell leukemia [73]. Another confirmed lncRNA was RRP1B (8th in the prediction results), which showed differential expression between acute myeloid leukemia patients with different treatments [74].

As mentioned above, many new lncRNA-disease associations have been added to the LncRNADisease database after June-2012 Version was publicly released. Therefore, I downloaded these new lncRNAs associated with colon cancer and leukemia and removed these lncRNAs which were not investigated in this study. Performance comparisons between IRWRLDA and LRLSLDA were implemented according to the rankings of these new disease-related lncRNAs in the case studies of colon cancer and leukemia (See Table 1). As a result, IRWRLDA significantly improved the prediction ability of LRLSLDA with higher ranks for these new disease-related lncRNAs.

**Table 1:** Performance comparisons between IRWRLDA and LRLSLDA based on the rankings of newly updated disease associated lncRNAs in LncRNADisease database for the Colon cancer and Leukemia

Disease	lncRNA	IRWRLDA	LRLSLDA
Colon cancer	CRNDE	1	32
Colon cancer	KCNQ1OT1	2	6
Colon cancer	MALAT1	3	3
Colon cancer	HOTAIR	4	15
Colon cancer	LSINCT5	52	115
Leukemia	MEG3	1	4
Leukemia	WT1-AS	2	110
Leukemia	DLEU2	4	10
Leukemia	CDKN2B-AS1	6	2
Leukemia	MIR155HG	12	71
Leukemia	DLEU1	18	63
Average ranks		9.55	39.18

### Applicability of IRWRLDA to diseases without any known related lncRNAs

In order to fully demonstrate that IRWRLDA could be effectively applied to human diseases without any known related lncRNAs, further case studies for these colon cancer and leukemia were implemented by removing all the known lncRNAs associated with investigated disease. In this validation framework, all the diseases don't have any known associated lncRNAs. In the lncRNADisease database, there are fourteen lncRNA-disease associations related with these four diseases after removing lncRNAs which were not investigated in this study. Therefore, IRWRLDA and LRLSLDA were implemented and the ranks of these seven associations were observed for each disease, respectively. As a result, it can be observed that IRWRLDA always provide higher rankings for these associations than LRLSLDA (See Table 2).

**Table 2:** Performance comparisons between IRWRLDA and LRLSLDA based on the rankings of fourteen lncRNA-disease associations related with colon cancer and leukemia

Disease	lncRNA	IRWRLDA	LRLSLDA
Colon cancer	CRNDE	1	35
Colon cancer	MALAT1	2	7
Colon cancer	HOTAIR	3	11
Colon cancer	KCNQ1OT1	4	52
Colon cancer	H19	6	1
Colon cancer	MEG3	8	4
Colon cancer	LSINCT5	21	92
Leukemia	MEG3	1	4
Leukemia	WT1-AS	2	49
Leukemia	DLEU2	4	13
Leukemia	CDKN2B-AS1	6	2
Leukemia	MIR155HG	13	75
Leukemia	DLEU1	18	74
Leukemia	TCL6	20	102
Average ranks		7.79	37.21

Here, case studies were implemented by removing all the known lncRNAs associated with investigated disease to fully demonstrate that IRWRLDA could be effectively applied to human diseases without any known related lncRNAs.

Leukemia is a group of cancers, including acute lymphoblastic leukemia (ALL), chronic lymphocytic leukemia (CLL), acute myeloid leukemia (AML), and chronic myeloid leukemia (CML) and so on. Each disease has its own pathological and mechanism properties and deserves to be investigated separately. Therefore, further case studies for these four kinds of important leukemia were implemented by removing all the known lncRNAs associated with investigated disease. As a result, it can also be observed that IRWRLDA significantly improve the predictive performance of LRLSLDA (See Table 3).

**Table 3:** Performance comparisons between IRWRLDA and LRLSLDA based on the rankings of seven lncRNA-disease associations related with ALL, CLL, AML, and CML

Disease	lncRNA	IRWRLDA	LRLSLDA
AML	MEG3	2	4
AML	WT1-AS	35	92
CML	MEG3	2	6
ALL	CDKN2B-AS1	13	2
CLL	DLEU2	1	14
CLL	DLEU1	71	117
CLL	MIR155HG	59	118
Average ranks		26.14	50.43

Here, case studies were implemented by removing all the known lncRNAs associated with investigated disease to fully demonstrate that IRWRLDA could be effectively applied to human diseases without any known related lncRNAs.

## DISCUSSION

Biological experiments have been the main methods to identify disease-lncRNA associations all the time. However, with an increasing number of newly discovered lncRNAs each year, experiment-based identification methods encounter many bottlenecks. Fortunately, a vast amount of lncRNA-related biological datasets in various publicly available databases make computational prediction feasible. In this study, the model of IRWRLDA was developed to effectively identify potential lncRNA-disease associations. First, lncRNA expression similarity, disease semantic similarity, and known lncRNA-disease associations were integrated to jointly decide the initial probability vector of the RWR. Furthermore, RWR would be implemented on the lncRNA similarity network constructed based on lncRNA functional similarity and lncRNA Gaussian interaction profile kernel similarity. IRWRLDA could be successfully applied to new diseases without any known associated lncRNAs. IRWRLDA significantly improved previous classical models in the validation framework of LOOCV based on two versions of known lncRNA-disease association datasets. In addition, 60% of the top 10 disease-lncRNA associations based on the prediction results of IRWRLDA were confirmed by recent biological experiment literatures.

IRWRLDA has demonstrated its reliable prediction performance in both cross validation and case studies of colon cancer and leukemia. The reliable performance could be largely attributed to four key factors listed as follows. Firstly, experimentally confirmed lncRNA-disease associations were obtained from highly reliable database and used as the training samples to identify the novel associations between lncRNAs and diseases. Secondly, various types of biological datasets were integrated to quantify the similarity between lncRNAs or diseases, including lncRNA functional similarity, lncRNA expression similarity, and disease semantic similarity. This data integration could effectively decrease the prediction bias. Furthermore, global network similarity measure was adopted in this study, whose advantage over local similarity measure-based models has been fully demonstrated in plenty of previous studies. More importantly, there are essential differences between IRWRLDA and traditional RWR. When the given disease is investigated, traditional RWR sets the initial probability vector only based on known related lncRNAs with this disease. Therefore, when this disease has no known related lncRNAs, RWR cannot work. Compared with traditional RWR, known lncRNA-disease associations, lncRNA expression similarity, and disease semantic similarity were combined to decide the initial probability vector of the RWR in the model of IRWRLDA, which ensures that IRWRLDA could be used to predict related lncRNAs for the diseases without any known related lncRNAs. Therefore, the application scope of classical RWR was significantly broadened.

However, there are also some limitations existing in the current version of IRWRLDA. For example, how to obtain integrated lncRNA similarity based on lncRNA functional similarity and lncRNA Gaussian interaction profile kernel similarity mentioned could be further improved. Furthermore, RWR was implemented on lncRNA similarity network constructed based on lncRNA functional similarity and lncRNA Gaussian interaction profile kernel similarity, while lncRNA expression similarity was used to decide the initial probability of candidate lncRNAs without known associations with the investigated disease. Among various lncRNA similarity measures, an effective approach need to be further developed to select which kinds of data sources for constructing lncRNA similarity network and other kinds of data sources to decide lncRNA initial probability. Furthermore, IRWRLDA may cause bias to lncRNAs with more known associated diseases or/and lncRNAs with more known interacting miRNAs or/and lncRNAs with miRNA interaction partners which has been associated with more diseases. However, heterogeneous biological datasets have been integrated in the model of IRWRLDA, which could not only effectively improve the prediction accuracy, but also decrease the prediction bias. More importantly, although IRWRLDA has significantly improved previous classical models, the room for improvement is still large. Finally, designing personalized computational models for disease-related lncRNAs identification and using personalized lncRNA biomarkers for different patients would deserve further investigation in the future [75–78].

## MATERIALS AND METHODS

### LncRNA-disease associations

Two versions of known lncRNA-disease association datasets (June-2012 Version and June-2014 Version) were downloaded from the LncRNADisease database [3], which was regarded as the gold standard in the cross validation (see Supplementary Tables S1 and S2). After removing the duplicate associations with different evidences, there were 293 and 621 distinct experimentally confirmed lncRNA-disease associations in these two datasets, respectively.

### Disease semantic similarity

Disease semantic similarity matrix *SS* was constructed, where *SS(i,j*) in row *i* column *j* indicated the semantic similarity between disease *d(i)* and *d(j)*, calculated based on their disease MeSH descriptors and DAGs [52]. Taking Disease *A* as an example, *DAG(A) = (D(A), E(A))* could be constructed, where *D(A)* is a node set consisting of the nodes of disease *A* itself and its ancestor diseases, and *E(A)* is an edge set composed of the direct edges from parent nodes to child nodes. In the traditional disease semantic similarity calculation model [52], disease terms in the same layer would have the same contribution to the semantic value of disease *A*. Actually, it is inaccurate to assign the same contribution value to two disease terms located in the same layer when they do not appear in the disease DAGs with the same frequency. In this work, the contribution of disease term *t* to the semantic value of disease *A* was calculated based on the assumption that a more specific disease term should have a greater contribution to the semantic value of disease *A*.

$$C_{A}\left(t\right)=-\log\left[\text{the number of DAGs including t}/\text{the number of diseases}\right]$$ (1)

The sematic value of disease *A* was obtained by summing up the contribution from all the disease terms in the *DAG(A)*.

$$C\left(A\right)=\sum_{t\in \text{DAG}\left(A\right)}C_{A}\left(t\right)$$ (2)

The semantic similarity between disease *A* and *B* can be calculated by summing the contributions of disease terms shared by these two diseases DAGs:

$$\text{SS}\left(A,B\right)=\frac{\sum_{t\in D\left(A\right)\cap D\left(B\right)}\left(C_{A}\left(t\right)+C_{B}\left(t\right)\right)}{C\left(A\right)+C\left(B\right)}$$ (3)

### LncRNA expression similarity

LncRNA expression similarity matrix *ES* was further constructed, where *ES(i,j)* was the lincRNA expression similarity between lncRNAs *l(i)* and *l(j)* when they were both lincRNA, otherwise 0. Based on the fact that comprehensive expression data of lncRNA are still unavailable so far and lincRNA occupies a large part of all the lncRNAs, lincRNA expression profiles were downloaded from UCSC Genome Bioinformatics (http://genome.ucsc.edu/) in October, 2012, including 21626 lincRNAs' expression profiles in 22 human tissues or cell types (Supplementary Table S3). LincRNA expression similarity was obtained by calculating the Spearman correlation coefficient between the expression profiles of each lincRNA pair.

### LncRNA functional similarity

Based on the assumption that lncRNAs with similar function tend to interact with similar miRNAs and similar miRNAs tend to be associated with similar diseases, the model of LFSCM calculates lncRNA functional similarity by integrating disease semantic similarity, miRNA-disease associations, and lncRNA-miRNA interactions in the previous study [49]. By this way, lncRNA functional similarity matrix *FS* was constructed, where *FS(i,j)* is the functional similarity between lncRNA *l(i)* and *l(j)*.

### LncRNA gaussian interaction profile kernel similarity

Based on the assumption that similar lncRNAs shows similar interaction and non-interaction pattern with the diseases [48, 79], lncRNA Gaussian interaction profile kernel similarity matrix *KL* was obtained based on known lncRNA–disease associations, where *KL(i,j)* was the Gaussian interaction profile kernel similarity between lncRNA *l(i)* and *l(j)* defined as follow:

$$\text{KL}(l(i),l(j))=\exp(-\gamma_{l}{\left\|\text{IP}(l(i))-\text{IP}(l(j))\right\|}^{2})$$ (4)

$$\gamma_{l}=\gamma_{l}^{'}/(\frac{1}{\text{nl}}\sum_{i=1}^{\text{nl}}\left|\left|\text{IP}\left(l\left(i\right)\right)\right|{|}^{2}\right)$$ (5)

where *IP(l(i))* and *IP(l(j))* are the binary vectors to encode whether known associations exist between lncRNA *l(i), l(j)* and each disease, respectively. The parameter *γ_l_* was denoted to control the kernel bandwidth and obtained through the normalization of a new bandwidth parameter *γ'_l_* by the average number of associations with diseases for all the lncRNAs.

### LncRNA integrated similarity

Based on lncRNA functional similarity and lncRNA Gaussian interaction profile kernel similarity mentioned above, lncRNA integrated similarity matrix *LS* was obtained as follows with trivial combinatorial coefficients, where *LS(i,j)* is the integrated similarity between lncRNA *l(i)* and *l(j)*.

$$\text{LS}(i,j)=\{\begin{array}{cc} \frac{\text{FS}(i,j)+\text{KL}(i,j)}{2} & i,j\in \text{IF}\\ \text{KL}(i,j) & \text{otherwise} \end{array}$$ (6)

where *IF* is the set of lncRNAs with corresponding lncRNA functional similarity based on the model of LFSCM [49].

### IRWRLDA

Based on the assumption that lncRNAs with similar functions tend to be associated with similar diseases [3, 48], the model of IRWRLDA was developed to predict potential disease-lncRNA associations (see Figure 1). So far, classical RWR has been successfully applied to computational biology researches [53, 54, 59–61, 63, 64]. However, considering the fact that traditional RWR set the initial probability vector only based on known related lncRNAs with this disease, so it cannot be applied to the diseases without any known associated lncRNAs. The essential difference between IRWRLDA and RWR mainly lies in the initial probability of lncRNAs. IRWRLDA consists of the following three steps: (1) decide the initial probability of all the lncRNAs, (2) implement random walk on the lncRNA similarity network based on integrated lncRNA similarity, and (3) obtain stable probability of random walk and rank candidate lncRNAs (see Figure 1).

For any given disease *d*, all the known associated lncRNAs were set to have the initial probability of 1. For the other lncRNAs, the maximums of two scores defined below, *score1* and *score2*, were defined as their initial probabilities by considering lncRNA expression similarity and disease semantic similarity. Firstly, the expression similarity between this lncRNA and lncRNAs which have known associations with disease *d* was calculated. Taking lncRNA *l* as an example, the formula was defined as follows:

score1(1)=Eli∈L(d)maxS(l,li) (7)

where *L*(*d*) is composed of all the lncRNAs having known associations with the disease *d*.

Then, the disease semantic similarity between disease *d* and all the diseases which have been confirmed to be associated with lncRNA *l* was further calculated based on the following formula.

score2(1)=Sdj∈D(l)maxS(d,dj) (8)

where *D*(*l*) is composed of all the diseases having known associations with the lncRNA *l*.

Finally, the initial probability of lncRNA *l* was obtained.

$$p_{0}\left(1\right)=\max(\text{score}1\left(1\right),\text{score}2\left(1\right))$$ (9)

where *p*_0_ is the initial probability vector.

In the second step of IRWRLDA, random walk was implemented on the lncRNA similarity network by starting at each lncRNA with the initial probability and transiting from current nodes to neighbors in the network based on the weight of the edge (i.e. the integrated similarity between these two lncRNAs). IRWRLDA allows the restart of random walk in each step with probability *r*. The column-normalized lncRNA integrated similarity matrix was denoted as *W*. Furthermore, the probability vector was defined as *p_t_*, where the *i*-th element is the probability of finding the random walk at node *i* at step *t*. Therefore, random walk was implemented according to the following iteration equation.

$$p_{t+1}=\left(1-\text{r}\right)\text{W}p_{t}+\text{r}p_{0}$$ (10)

After some steps, stable probability *p*_∞_ could be obtained when the change between *p_t_* and *p*_*t* + 1_ is less than a given cutoff based on *L*_1_ norm. All the candidate lncRNAs were ranked according to *p*_∞_ and lncRNAs with high scores were expected to be potentially related with investigated disease *d*.

## SUPPLEMENTARY MATERIALS TABLES








