# Supplementary material for: IRWRLDA: improved random walk with restart for lncRNA-disease association prediction
Source: Oncotarget. 2016 Aug 9;7(36):57919–31. doi: 10.18632/oncotarget.11141 (PMC5295400; doi:10.18632/oncotarget.11141)
Supplement: Supplementary file 1 [file oncotarget-07-57919-s001.pdf]

IRWRLDA: improved random walk with restart for lncRNA-disease association prediction

Supplementary Materials

Supplementary Table S1: Known lncRNA-disease association datasets (June-2012 Version) were downloaded from the LncRNADisease database, which was regarded as the gold standard in the cross validation. See Supplementary_Table_S1

Supplementary Table S2: Known lncRNA-disease association datasets (June-2014 Version) were downloaded from the LncRNADisease database, which was regarded as the gold standard in the cross validation. See Supplementary_Table_S2

Supplementary Table S3: LincRNA expression profiles were downloaded from UCSC Genome Bioinformatics (http://genome.ucsc.edu/) in October, 2012, including 21626 lincRNAs′ expression profiles in 22 human tissues or cell types. See Supplementary_Table_S3
